# Strategies for Improving Contact-Electro-Catalytic Efficiency: A Review

**DOI:** 10.3390/nano15050386

**Published:** 2025-03-02

**Authors:** Meng-Nan Liu, Jin-Hua Liu, Lu-Yao Wang, Fang Yin, Gang Zheng, Ru Li, Jun Zhang, Yun-Ze Long

**Affiliations:** 1 Collaborative Innovation Center for Nanomaterials & Devices, Innovation Institute for Advanced Nanofibers, College of Physics, Qingdao University, Qingdao 266071, China; 2 Instrumental Analysis Center of Qingdao University, Qingdao 266071, China; 3 State Key Laboratory of Bio-Fibers and Eco-Textiles, Qingdao University, Qingdao 266071, China

**Keywords:** contact-electro-catalysis (CEC), degradation, ultrasonication, catalytic efficiency

## Abstract

Contact-electro-catalysis (CEC) has emerged as a promising catalytic methodology, integrating principles from solid-liquid triboelectric nanogenerators (SL-TENGs) into catalysis. Unlike conventional approaches, CEC harnesses various forms of mechanical energy, including wind and water, along with other renewable sources, enabling reactions under natural conditions without reliance on specific energy inputs like light or electricity. This review presents the basic principles of CEC and discusses its applications, including the degradation of organic molecules, synthesis of chemical substances, and reduction of metals. Furthermore, it explores methods to improve the catalytic efficiency of CEC by optimizing catalytic conditions, the structure of catalyst materials, and the start-up mode. The concluding section offers insights into future prospects and potential applications of CEC, highlighting its role in advancing sustainable catalytic technologies.

## 1. Introduction

Catalysis can initiate or accelerate chemical reactions without altering chemical equilibrium, which plays an important role in the fields of chemical synthesis, industrial production, biomedical, energy and environment [1,2,3,4]. Catalytic efficiency can be effectively improved by selecting new catalysts, optimizing catalyst structures, improving catalyst purity, and controlling catalytic reaction conditions, which can not only improve production efficiency, reduce costs, and save energy, but also protect the environment, promote technological innovation, and drive the development of new energy. Polymer catalysts have loose reaction conditions, high efficiency, easy recycling, and reusability, but their stability is poor and their design and synthesis are complex. Inorganic nanoparticles have a high specific surface area and abundant active sites, and exhibit good catalytic stability, which can significantly improve the activity and selectivity of catalytic reactions. However, they are not easy to separate, recycle, and reuse, causing problems such as resource waste and environmental pollution. Polymer nanocomposites (PNCs) obtained by combining polymers and inorganic nanoparticles have the advantages of both, with significant mechanical properties and compatibility, providing possibilities for solving their respective problems. Based on the advantages of PNCs as environmental remediation catalysts, Tushar et al. [5] first discussed the catalytic process and mechanism of pollutant degradation of PNCs, then detailed the applications of PNCs in catalytic and redox degradation, electrocatalytic degradation, and biocatalytic degradation of pollutants, and finally comprehensively summarized the challenges and prospects of PNCs in the field of environmental remediation. Traditional catalytic methods rely on the physical and chemical properties of catalysts to promote chemical reactions. Currently, the primary catalytic methods used in practical applications include photocatalysis, electrocatalysis, piezoelectric catalysis, tribocatalysis, and thermal catalysis [6,7,8,9,10]. These methods require catalysts to possess certain characteristics, such as thermal conductivity, electrical conductivity, or photosensitivity. Photocatalysis accelerates chemical reactions by generating electron-hole pairs on surface upon light excitation [11,12]. Typical materials used for photocatalysis include metal oxides, and doped composite semiconductor materials, etc. [13,14]. Although solar energy is clean, it is limited by lighting conditions and poor transparency through dark dye solutions hinder its full potential. On the other hand, electrocatalysis mainly uses noble metal materials and utilizes externally applied electric fields to catalyze the degradation or conversion of organic pollutants [15,16]. However, its dependence on an external high electric field necessitates a significant energy investment. Traditional preparation methods are difficult to control the distribution of active components, pore structure, and pore size, with low automation and high labor intensity, which not only reduces the activity or failure of the catalyst, but also affects its production efficiency and quality control. Moreover, traditional catalysts are prone to failure during use, and the regeneration process is not thorough, which affects the reuse effect and lifetime of the catalyst. In addition, there is a problem of large loss during the separation process of catalysts, resulting in low utilization and increased production costs. All of these greatly limit the development of traditional catalytic methods in practical applications. To address these challenges, researchers are exploring fossil energy-independent catalytic technologies such as mechanical catalysis, to generate reactive oxygen species (ROS) for catalytic applications. Mechanical technologies catalysis, including piezoelectric catalysis, tribocatalysis, and contact-electro-catalysis (CEC), can directly utilize mechanical energy for catalysis, without complex energy inputs [17]. The catalyst used for piezoelectric catalysis must have piezoelectric properties, and there are certain limitations in the choice of materials. Different from traditional catalytic methods, CEC is a novel catalytic mechanism utilizing electric field regulation to achieve catalytic reactions proposed by Wang et al. in 2022 [17]. By applying an electric field on the electrode surface, the interaction between the catalyst and reactants is regulated, thereby triggering catalytic reactions at the active sites. This mechanism makes the transfer of electrons and ions more efficient, reducing the occurrence of side reactions and thus improving the catalytic efficiency. It participates in redox reactions through electron transfer caused by the contact-electrification (CE) between solid and liquid, or liquid and liquid interfaces [18].

The introduction of CEC represents a paradigm shift in catalytic degradation using solid-liquid triboelectric nanogenerators (SL-TENGs). CEC harnesses electron transfer induced by contact-separation phenomena between liquid and solid interfaces, initiating or accelerating chemical reactions without conventional catalysts. Unlike traditional photocatalytic and electrocatalytic materials that tend to deactivate during reactions, CEC employs polymers with stable electronic structures and dielectric materials with strong electron-gaining abilities, expanding catalyst options [18]. Most research conducted by CEC has focused on treating organic wastewater. In the past two years, researchers have successively developed more application areas, such as metal reduction and H_2_O_2_ synthesis. CEC can fully utilize mechanical energy from the environment, but compared to electrocatalysis and photocatalysis, it has not yet achieved large-scale industrial application due to its conversion rate in energy and catalysis. Compared with traditional catalytic methods, CEC utilizes electron transfer generated by contact electrification under mechanical excitation to complete catalytic reactions. It is not limited by the conductivity of the reactant surface, and as long as the material can contact electrification, catalytic reactions can be realized. For example, commercial organic polymers such as FEP and PTFE [17,19,20,21,22], as well as inorganic materials such as Bi_2_WO_6_, LiNbO_3_, BaTiO_3_, nZVC-CNT/PMS, and CDs/ZnO [23,24,25,26,27], can all produce reactive oxygen species (ROS) through CEC catalysis. Therefore, it greatly expands the selection range of catalysts and provides more diverse possibilities for catalytic system design. Furthermore, compared to the local reaction effects determined by UV irradiation, electrical energy input, and other methods, the reaction range of CEC is more comprehensive and has greater prospects for large-scale applications [18]. Moreover, the catalytic efficiency of CEC is comparable and even superior to that of the existing mechanochemical strategies, but it is relatively sluggish in comparison with traditional catalytic methods such as electrocatalysis or photocatalysis. In order to effectively improve the catalytic efficiency of CEC, existing materials can be surface modified or structurally optimized to enhance their CE ability, and new materials can also be developed to explore novel start-up methods. Thanks to the almost unlimited selection of catalysts by CEC, a large number of environmentally friendly materials can be used for catalysis. And, these catalysts and substrates can be efficiently separated through simple methods, avoiding secondary pollution to the environment. Meanwhile, these catalysts can be repeatedly recycled and reused, further reducing the environmental pollution caused by the preparation process. In addition, due to the widespread occurrence of contact electrification effects between various materials such as solids and solids, solids and liquids, solids and gases, and liquids and gases, CEC can not only be used to catalyze water splitting to produce hydrogen gas for clean energy production, but also to efficiently catalyze organic wastewater to reduce environmental pollution. Therefore, CEC will lead a series of cutting-edge catalytic research, providing new principles and ideas for solving a series of national strategic and livelihood issues such as carbon neutrality, new energy, water resources, pharmaceuticals and chemicals. To further enhance the catalytic efficiency of CEC, researchers are conducting various theoretical and experimental explorations. Based on past experience, the occurrence conditions and material properties are important influencing factors of CEC. Wang et al. have theoretically and experimentally verified the influence of surface defects on the efficiency of CEC, demonstrating that the addition of surface functional groups can enhance the rate of MO degradation by FEP [28]. Moreover, the carrier separation promotion strategy based on polarized internal electric fields can effectively improve the performance of ferroelectric materials in photocatalysis and piezocatalysis. Li et al. [29] established the internal electric field of CEC catalyst by charging it through an electret process using polytetrafluoroethylene (PTFE) medium. The electron transfer barrier between the PTFE and the water atoms was reduced by 37% under the internal electric field. And methyl orange achieved a degradation rate of over 90% after 1.5 h of catalysis. The results demonstrate the feasibility of optimizing the electret strategy of catalysts in improving catalyst efficiency. We still need to continuously explore in depth how to improve the catalytic efficiency of CEC in order to achieve regulation of catalytic reaction pathways in the future.

**Figure 1:**
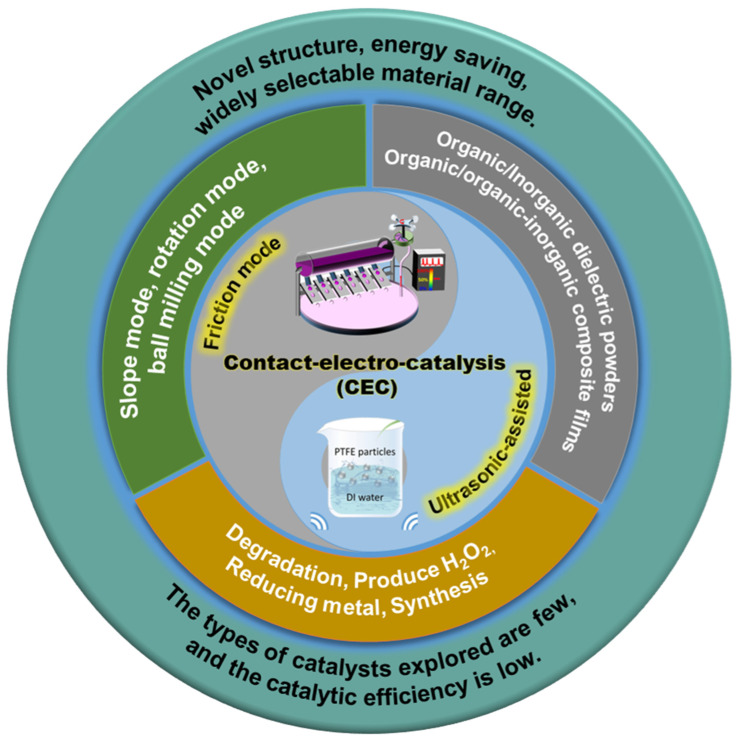
The modes of occurrence, catalyst material, applications of CEC, and advantages and disadvantages. Reproduced with permission from [20], Adv. Mater.; published by Wiley, 2023. Reproduced with permission from [30], Water Res.; published by Elsevier, 2022.

This paper comprehensively reviews the research progress of CEC in recent years (Figure 1), introduces its basic principles, characterization techniques, and theoretical calculations, and discusses the limitations of traditional catalytic methods and the advantages of CEC. In addition, this article summarizes the current applications of CEC in the degradation of organic dyes and molecules, preparation of H_2_O_2_, recovery of metals and synthesis of chemical substances. At the same time, this paper evaluates the catalytic efficiency of CEC, especially analyzes the current strategies used to improve its catalytic efficiency, mainly including changing the environmental conditions of catalysis, optimizing the structural design of materials and adjusting the initiation mode of the catalytic. Finally, this paper reviews the potential applications of CEC in environmental protection and energy conservation, providing research ideas and directions for the practical application of CEC in large-scale industries.

## 2. Proposal and Fundamental Principles of CEC

### 2.1. TENGs and Catalysis

**Figure 2:**
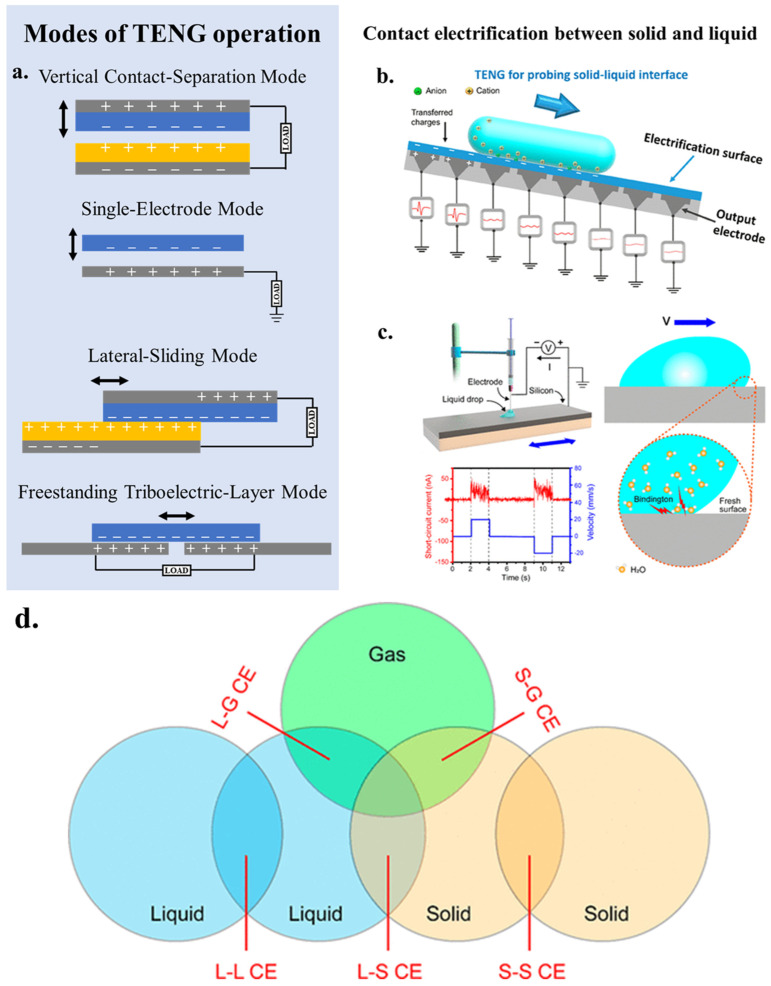
The four basic modes of TENG. (**a**) Vertical contact-separation mode; Lateral-sliding mode; Single-electrode mode; Freestanding triboelectric-layer mode. Reproduced with permission from [33], Adv. Energy Mater.; published by Wiley, 2019. (**b**) SL-TENG for probing solid-liquid interface. Reproduced with permission from [34], Chem. Rev.; published by ACS, 2022. (**c**) Generation of tribovoltaic effect at the sliding water and semiconductor interface. Reproduced with permission from [35], Nano Energy; published by Elsevier, 2020. (**d**) Schematic of the contact electrification among the interface of solid, liquid and gas.

As an energy conversion technology, triboelectric nanogenerators can convert mechanical energy into electrical energy. In 2012, Wang’s group proposed the concept of triboelectric nanogenerators, which relies on the combination of triboelectric effect and electrostatic induction [31]. When materials with differing electronegativities come into contact or are rubbed together under external force, they develop positive and negative electrostatic charges on their surfaces. Upon separation, these charges separate, creating a potential difference that drives electron flow between the upper and lower electrodes, generating electric current. TENGs possess the ability to harness untapped mechanical energy from the environment, such as irregular low-frequency and low-amplitude mechanical energy, converting it into electrical energy, thereby enhancing overall energy utilization [32]. In recent years, various modes of TENG operation have emerged, including vertical contact-separation mode, single-electrode mode, lateral-sliding mode and freestanding triboelectric-layer mode (Figure 2a) [33]. The traditional TENGs are contact electrification (CE) at the interface of two solid materials, while CE between solid and liquid interfaces expands the application of TENGs in catalysis. Combined by vertical contact-separation mode and single-electrode mode, we can stimulate the catalytic reaction by ultrasonication, ball milling, slope sliding and rotation [34] (Figure 2b). In addition, chemical reactions can be initiated by the lateral-sliding of the liquid across the solid interface and by stirring [34,35] (Figure 2c). Contact-separation is a key process in CEC, and it has been proven that CE can occur between solid, liquid, and gas states (Figure 2d) [34]. Therefore, we can boldly speculate that any solid-gas interface, liquid-liquid interface, gas-liquid interface, etc. that can undergo CE can be used for catalysis.

**Figure 3:**
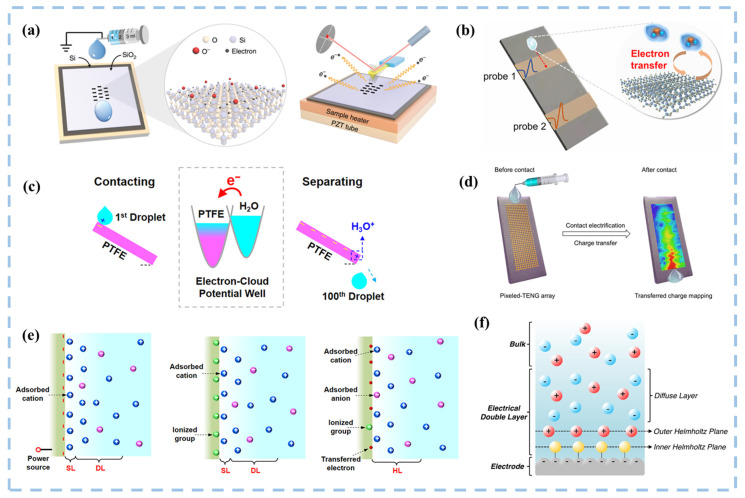
Exploring the microscopic mechanism of charge transfer during CE at solid-liquid interfaces. (**a**) AFM platform for thermionic emission experiments to verify the CE charge on SiO_2_ surface at different substrate temperatures. Reproduced with permission from [37], Nat. Commun.; published by Nature, 2020. (**b**) Measurement of the current signal of a drop flowing over a polymer surface by two Cu electrodes, respectively. Reproduced with permission from [38], ACS Nano; published by ACS, 2021. (**c**) Electron-cloud potential wall diagram of a droplet sliding down a polymer ramp. Reproduced with permission from [40], ACS Nano; published by ACS, 2020. (**d**) Measure the induced charge at each point where the water droplet slips through the electrode array. Reproduced with permission from [39], ACS Nano; published by ACS, 2023. (**e**) ‘Wang’s hybrid layer’ model. (**f**) Schematic of the electrical double layer. Reproduced with permission from [57], Curr. Opin. Chem. Eng.; published by Elsevier, 2018.

The electron transfer process caused by contact-electrification between solid-liquid interfaces involves material surface and interface science, including many physical and chemical mechanisms that require in-depth exploration in order to better select and optimize triboelectric materials [36]. As illustrated in Figure 3, Wang’s research team employed diverse probes to investigate the microscopic mechanism of charge transfer in solid-liquid contact electrification [37,38,39]. They further proposed “Wang’s hybrid layer model”, a mixed-layer model at the interface between a liquid and a solid polymer, which incorporates cations attracted by transferred electrons, ionizing groups formed on the surface of solids, and anions held together by van der Waals forces. This model underscores electrons as the primary species facilitating charge transfer across the liquid-solid interface [40], suggesting the potential for catalyzing chemical reactions. SL-TENGs are used to harvest ocean blue energy on a large scale, efficiently collecting energy from low-frequency vibrations and producing high voltages as output [36,41,42]. SL-TENGs are currently offered in several configurations, which are classified into droplet-type [43,44], flow-type [45], wave-type [46], and other novel modes [47]. Its applications span diverse fields, encompassing micro/nano-energy and blue energy harvesting [48], flexible wearable self-powered sensing systems [49,50,51,52], medical applications [53], and environmental monitoring [54], among others [55]. Numerous forms of nanogenerators have also been developed to cater to different application fields. However, conventional TENGs typically generate alternating current (AC), and the friction between two solid materials can be highly abrasive. To broaden their applications and improve the durability of the friction layer, innovative TENGs capable of generating direct current (DC) [56] and employing solid-liquid interface friction have been introduced.

A new way of catalyzing degradation using SL-TENGs, called contact-electro-catalysis (CEC), was proposed by Wang’s team in 2022 [17]. CEC operates as a form of tribocatalysis. During the liquid-solid contact-separation process, electron exchange occurs at their interfaces, known as contact-electrification, leading to the generation of strong oxidizing free radicals in the solution, thereby facilitating the oxidation and decomposition of organic pollutants. Unlike traditional photocatalytic and electrocatalytic materials that tend to deactivate during reactions, CEC employs polymers with a stable electronic structure and dielectric materials with strong electron-gaining abilities directly in catalysis, thus broadening the range of available catalysts and degradation pathways. CE, being ubiquitous across various interfaces, allows for the design of a new class of catalysis by integrating clean energy sources such as wind, water, and solar energy, contributing to energy conservation and environmental protection [17]. Most catalyst materials in CEC are highly electronegative insulating materials, typically fluorine-containing polymers or those treated with fluorine modification. In the context of solid-liquid electron transfer, polymers like PTFE and FEP usually act as electron acceptors, while water serves as the electron donor [58].

**Figure 4:**
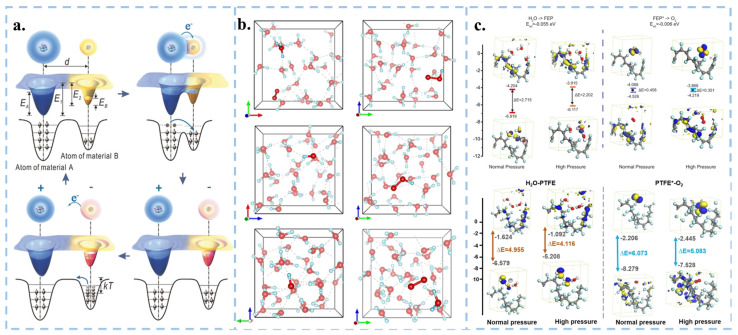
Theoretical calculations of electron transfer in solid-liquid contact. (**a**) Electron-cloud-potential-well model. Reproduced with permission from [59], Adv. Mater.; published by Wiley, 2018. (**b**) AIMD simulation in aqueous solution at different pressures. Reproduced with permission from [21], Nanoscale; published by RSC, 2023. (**c**) DFT calculations of the values of LUMO and HOMO levels for H_2_O-Solid and O_2_-Solid in various conditions. Reproduced with permission from [17], Nat. Commun.; published by Nature, 2022. Reproduced with permission from [20], Adv. Mater.; published by Wiley, 2023.

CEC harnesses the SL-TENG principle for catalysis, primarily targeting the degradation of organic wastewater. Driven by external mechanical energy, the catalyst and solution undergo repeated cycles of contact-separation, facilitating electron transfer between air, aqueous solution, and solid catalyst, thus promoting chemical reactions. The generation of ROS through the contact-separation of the catalyst and aqueous solution can be broadly divided into two processes. Firstly, when a water molecule comes into contact with a solid catalyst denoted by *S*, the electrons in the water molecule are transferred to the catalyst (as described in Equation (1)). This process constitutes CE between the solid and the liquid, arising from the collision between water molecule and solid within the repulsion region, wherein electron transfer occurs due to the overlap of their electron clouds [59,60], as described in Figure 4a. As cations in the solution accumulate on the surface of S*, an electrostatic double layer (EDL) is formed [34,61]. The water molecule that loses one electron in solution combines with another water molecule to generate ·OH and hydronium ions ($H_{3}O^{+}$) (as expressed in Equation (2)). Simultaneously, the solid catalyst gains an electron from the water molecule, symbolized as S*. A fraction of *O*_2_ dissolves in the water, and influenced by the energy supplied by visible light, heat, and phonons, electrons on the surface of the solid catalyst (S*) become excited, combining with *O*_2_ to form ${\cdot O}_{2}^{-}$ (Equations (3) and (4)) [62]. Meanwhile, the electron exchange between S* and *O*_2_ can also occur (Equation (5)), leading to the formation of radicals that can subsequently react with $H_{3}O^{+}$ to form ${HO}_{2}^{\cdot }$ (Equation (6)) [17]. Hence, CEC can exert various effects across different interface environments, including but not limited to dye degradation, hydrogen peroxide synthesis, electrode recovery, and nitrogen reduction.

(1) $$H_{2}O+S\to H_{2}O^{+}+S^{*}$$

(2) $$H_{2}O^{+}+H_{2}O\to \cdot OH+H_{3}O^{+}$$

(3) $$S^{*}\overset{visible\;light,\;heat\;or\;phonons\;energy}{\to }e^{-}+S$$

(4) $$O_{2}+e^{-}\to {\cdot O}_{2}^{-}$$

(5) $$S^{*}+O_{2}\to {\cdot O}_{2}^{-}+S$$

(6) $${\cdot O}_{2}^{-}+H_{3}O^{+}\to H_{2}O+{HO}_{2}^{\cdot }$$

Further investigation into the detailed mechanism of CEC is warranted. The energy barriers for electron transfer when water and oxygen molecules interface with solids can be effectively estimated using density functional theory (DFT). Assume that localized high pressure and high heat are generated when cavitation bubbles burst in the solution under ultrasonic environment. By simulating a high-pressure environment through ab initio molecular dynamics (AIMD), the energy barrier for electron transfer at the solid-liquid and solid-gas interfaces is lowered when the system volume is compressed, making electron transfer more easily occur (Figure 4b,c). Accordingly, the process of solid-liquid contact electron transfer can be theoretically verified.

### 2.2. Mechanochemical Reactions

Mechanochemistry is the combination of mechanical processing and chemical reactions at the molecular level. At the level of chemical reactions, mechanical energy is mainly applied to condensed substances such as solids and liquids through methods such as shear, friction, impact, and compression, inducing changes in their structure and physical and chemical properties, and triggering chemical reactions. Mechanochemistry is the study of chemical reactions caused by mechanical energy [63]. The mechanochemical reaction is powered by mechanical energy and can be performed under various conditions, such as in low temperature, inert or active atmosphere, high pressure, solvent or all-solid state conditions. Therefore, mechanochemical reactions are simple, universal, and sustainable. In addition, unlike most chemical synthesis carried out in solution, mechanochemical reactions almost do not use solvents, making them relatively safe, clean, and efficient.

Mechanochemistry, as a way of converting mechanical energy into chemical energy, has recently received widespread attention. From a physical view, piezoelectric catalysis, tribocatalysis, and CEC all fall under the umbrella of mechanochemistry [64,65]. Mechanochemical reactions occur when mechanical energy is directly absorbed, through processes such as impact, tension, and friction, followed by ball-milling, pan-milling, and ultrasonic irradiation [66]. These reactions induce phase and structural changes, crystal transformations, decreased crystallinity, surface activation, and mechanochemical transformations. Additionally, thermal effects often accompanied by mechanical stress, thereby influencing reaction pathways. From a dynamic perspective, mechanochemical reactions can be elucidated through collision theory. The kinetic energy generated during collision processes facilitates wear, fracture, and refinement of the chemical system’s microstructure. Most importantly, the formation of new interfaces during the fracture process can significantly increase the specific surface area of the material, thereby increasing the probability of contact between different components. Due to the fact that reactions typically occur on the surfaces or boundaries of different components, chemical reactions are passively accelerated [67,68]. Moreover, experimental results obtained through mechanochemistry are often unpredictable, making mechanochemical reaction ideal candidates for exploring new chemical reactions. For example, Coleman et al. [69] obtained single-layer functional materials such as molybdenum disulfide (MoS_2_), tungsten disulfide (WS_2_), and molybdenum diselenide (MoSe_2_) through ultrasonic exfoliation.

Mechanochemistry is a promising method for synthesizing new materials, which has wide applications in the synthesis of inorganic and organic materials, promoting co-crystallization, preparing nanomaterials, metal complexes, and polymers [70]. Mechanochemical techniques such as ball milling, flat grinding, and ultrasonic irradiation have been successfully applied in the preparation of piezoelectric and frictional electrical devices for mechanical energy to electricity conversion [71], solar cells for light energy to electricity conversion [72], LEDs and electrochromic materials [73], as well as supercapacitors (SCs) for chemical energy to electricity conversion and storage [74] and advanced secondary batteries [75]. While there are already notable studies on mechanochemistry [76], partly focusing on CEC, providing enormous potential for further exploration and innovation in CEC.

### 2.3. Different Catalytic Methods

Catalysis refers to the process of reducing the activation energy of reactants through catalysts, thereby accelerating the rate of chemical reactions. Typically, employing the appropriate catalyst, which is generally unaffected by catalytic action itself, enables repeated or continuous catalytic activity. The overall efficacy of any catalyst hinges on the cumulative efficiencies of the reaction steps. Thus, assessing the rate-limiting step during these reaction steps, often linked to adsorption, reaction, and desorption on the catalytic surface, becomes paramount. The catalytic methods can be divided into photocatalysis, electrocatalysis, piezocatalysis, tribocatalysis, and CEC. Photocatalysis mainly utilizes semiconductor materials, which have cost advantages over precious metals, fostering electron-hole pair generation through charge carrier photoexcitation. This process, dependent on bandgap width, involves intricate dynamics of charge generation, separation, and transfer. Electrocatalysis mainly uses Pt and other precious metal materials. Electrocatalysis refers to the use of different interfaces (solid/liquid, liquid/liquid, solid/solid, mostly at the electrode/solution interface) charge transfer to promote the process of chemical reaction, in which the electrode as a catalyst, through the gain and loss of electrons to activate the reactant molecules, reduce the reaction activation energy, thereby accelerating the chemical reaction rate. A common feature of electrocatalysis is that the reaction process involves two or more consecutive steps [3]. Piezocatalysis harnesses piezoelectric materials’ mechanical deformation to induce piezoelectric potential, triggering the separation of intrinsic electrons and holes, and catalyzing electrochemical reaction [77,78]. Thermoelectric catalysis, an essential subset of piezoelectric catalysis, inherent in thermoelectric materials with internal polarization, exploits temporal temperature changes-induced charge separation to drive catalytic activity [79,80]. Tribocatalysis represents a novel paradigm in catalysis, harnessing the triboelectric effect generated by mechanical energy. In contrast to piezoelectric materials, which necessitate a specific crystal structure, tribocatalysis boasts a broader spectrum of applicable materials [81]. It operates through two distinct mechanisms: electron transition and electron transfer. Electron transition entails the generation of electron-hole pairs through mechanical energy, while electron transfer involves the friction between the catalyst and its surrounding solid or liquid environment, facilitating the direct transfer of electrons crucial for catalysis. Consequently, tribocatalysis typically instigates and expedites redox reactions through the collaborative interplay of highly electronegative materials and catalysts.

Irrespective of above catalytic processes, the catalytic material and electrolyte significantly influence the solid-electrolyte interface, impacting electron transfer kinetics and forming an immobilized charge layer known as the Helmholtz layer (Figure 3f). Modulation of surface potential directly affects the concentration of ionic species in the Helmholtz layer, influencing mass transfer properties and overall redox process kinetics. Differentiating between effects involving free charge carriers (photocatalysis and electrocatalysis) and those inducing bound surface charge density through polarization (piezocatalysis) is essential for deeply understand catalytic mechanisms, optimize energy conversion efficiency, and expand application fields [78]. Table 1 presents a compilation of commonly used catalysts along with the characteristics of photocatalysis, electrocatalysis, piezoelectric catalysis, and CEC, as well as their respective advantages and disadvantages. Compared with other catalytic methods, the occurrence of CEC is extremely convenient. As long as the material can come into contact with electricity, catalytic reactions can be achieved. Therefore, this greatly expands the selection range of catalysts and provides the possibility for designing more diverse catalytic systems. Moreover, compared to methods such as lighting and electrical energy input that determine local reaction effects, the reaction range of CEC is more comprehensive and has the potential for large-scale applications. In addition, CEC has the unique advantage of being green and environmentally friendly, thanks to its almost unlimited selection of catalysts. Therefore, a large number of environmentally friendly materials can be used for catalysis, and these catalysts can be efficiently separated from substrates in a simple way. This not only avoids secondary pollution to the environment, but also allows for repeated recycling and use. However, CEC currently has a limited variety of suitable catalysts, and the catalytic efficiency needs to be improved. Therefore, future research directions are expected to focus on exploring new catalysts that are non-secondary-polluting, recyclable, easy to recover, and can be seamlessly combined with clean energy to maximize catalytic efficiency.

## 3. Progress in Applied Research of CEC

CEC can generate ROS by different modes of mechanical motion (ultrasonication, friction, etc.) to degrade organic substances, produce H_2_O_2_, reduce metal and synthesize chemical substances. The materials used as catalysts are organic polymer powders, inorganic powders, organic polymer films and organic-inorganic composites. Ultrasonication is one of the most commonly used forms of CEC. Ultrasonic-assisted CEC processes involve immersing the solution and catalyst in an ultrasonic bath, subjecting them to frequent contact-separation induced by specific frequency and power settings. Cavitation arises from the nucleation of bubbles during the interaction between ultrasound and liquid. Depending on the frequency and power, cavitation can manifest as stable or inertia. Stable cavitation generates microfluidic flows within the water environment, converting sound energy into heat, while inertial cavitation leads to bubble expansion and collapse, reaching pressures of 40–60 kbar upon rupture [81]. These collapsing bubbles exert shear forces within the reaction mixture, driving mechanochemical reactions [82]. Frequency and power play a crucial role in determining the quantity and characteristics of cavitation bubbles. High ultrasound frequencies lead to an increased number of cavitation bubbles, albeit with a reduction in their size [83]. As frequency increases, the oscillation period of the bubbles shortens, making them more prone to collapse and fragmentation. The generation of free radicals in active substances relies on factors such as the collapse temperature and steam composition within the bubble [84,85]. Additionally, sound power supplies the necessary pressure for cavitation, with high power levels resulting in an augmentation of both the number and size of cavitation bubbles [86,87,88]. Forces from the sonic field are crucial for initiating the CEC cycle, enabling electron transfer and cavitation bubble formation. Upon bubble collapse, high-pressure microjets facilitate electron exchange with dissolved oxygen, collectively reducing the energy barrier for electron transfer and promoting ROS production.

### 3.1. CEC for the Degradation

**Figure 5:**
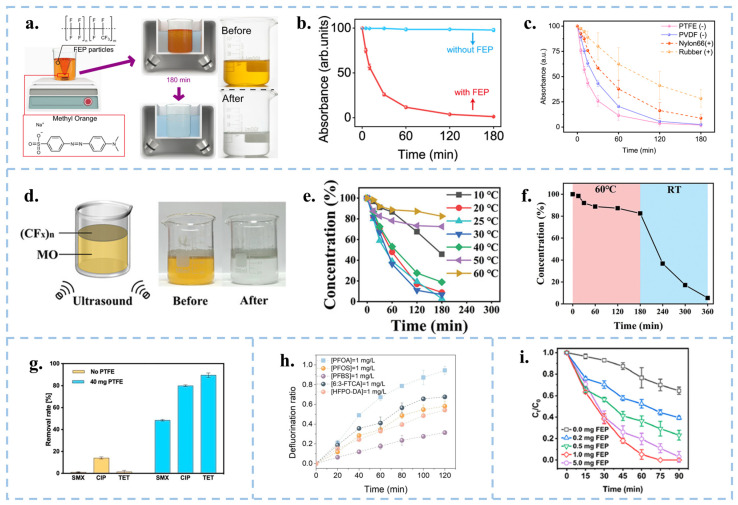
(**a**) Schematic diagram of the experimental device and scheme for the ultrasonic degradation of MO by FEP powder. (**b**) Changes in absorbance of MO solution with/without FEP powder. (**c**) Variation of UV-Vis absorbance of MO solution during ultrasonic treatment with PTFE, PVDF, Nylon-6,6 and NBR rubbers, respectively. Reproduced with permission from [17], Nat. Commun.; published by Nature, 2022. (**d**) Schematic diagram of the device for ultrasonic degradation of MO by fluorinated graphite. (**e**) Ultrasonic degradation of MO by fluorinated graphite concentration at different temperatures. (**f**) The catalytic efficiency of (CF_X_)_n_ was restored as before when the temperature was lowered from high to room temperature. Reproduced with permission from [89], Adv. Funct. Mater.; published by Wiley, 2024. (**g**) Removal rates of 1 mg/L SMX, CIP and TET solutions with/without PTFE particles. Reproduced with permission from [90], Chem. Eng. J.; published by Elsevier, 2024. (**h**) Defluorination of solutions of different PFAS compounds by ultrasonication of PTFE particles. Reproduced with permission from [91], Angew. Chem. Int. Ed.; published by Wiley, 2024. (**i**) Concentration changes in ultrasonic degradation of pentachlorophenol by different masses of FEP. Reproduced with permission from [92], ACS EST Eng.; published by ACS, 2024.

In 2021, Wang’s group explained that the contact electrification at the solid-liquid interface during contact-separation is mainly the exchange of electrons based on the principle of SL-TENGs. At the same time, they proposed a novel catalytic method of CEC and verified it through experiments. They successfully achieved the degradation of 50 mL, 5 ppm methyl orange (MO) solution by ultrasonic treatment of 20 mg fluorinated ethylene propylene (FEP) dielectric powder for 180 min [17] (Figure 5a,b). In addition, CEC is observed for various dielectric materials, such as FEP, PTFE, PVDF. However, those with a strong electron gaining ability are more easily to undergo CEC (Figure 5c). Conversely, when the catalyst exhibits opposite electrode properties to the target organic ions, significant physisorption occurs. The catalytic principles underlying these pristine dielectric materials not only broaden the selection of catalytic materials but also enable the catalytic process to be induced through mechanical induction. Ultrasonic treatment generates a lot of heat, and the suitable reaction temperature for these organic polymer catalysts is 20–30 °C. If the temperature is excessive, it will glassify, resulting in a loss of catalytic performance, which requires us to find catalysts suitable for working at high temperatures. Long et al. carried out contact electrocatalytic degradation of organics with an inorganic material ((CF_x_)_n_-2D fluorinated graphite) [89]. The material is thermally stable and does not deactivate at high temperatures, but the catalytic efficiency is still unsatisfactory. In this regard, they proposed that it was due to the weakening of the cavitation effect at high temperatures. The discovery of this material has opened up the application of two-dimensional inorganic materials in catalysis and gradually expanded the range of catalyst choices.

In addition to degrading organic dyes, Chen et al. [90] were able to degrade three typical antibiotics (sulfamethoxazole (SMX), ciprofloxacin (CIP)) and tetracycline (TET)) using PTFE pellets after 90 min of sonication with removal rates of 50%, 80%, and 90%, respectively (Figure 5g). Thus, the CEC reaction appears to be highly efficient for the degradation of quinolones and tetracyclines and relatively inefficient for sulfonamides. Jin et al. [91] similarly removed perfluoroalkyl substances (PFASs) from water by ultrasonication using PTFE particles (Figure 5h). Yuan et al. [92] utilized FEP powder ultrasound for effective removal of PCP (Figure 5i). CEC uses chemically inert materials to catalyze chemical reactions, expanding the range of catalysts. As a new direction, the theory of CEC still needs to be improved. The use of micro-nanoparticles as catalysts must consider the recyclability and reproducibility. Fluorine-containing polymers are highly surface-charged and chemically sluggish. They are stable in the environment for a long time and are not easily decomposed, which can cause secondary pollution. Therefore, it is necessary to find safe and non-polluting catalyst materials with high recyclability, reusability and degradation efficiency, which are also of great significance for large-scale industrial applications. Although all of the above literature suggests that the catalytically finished powders can be recovered, in reality, it is difficult to recover them completely. How to deal with the unrecovered powders is then a core issue that needs to be addressed when using powders for CEC. Film materials are capable of achieving complete recycling, but the degradation of finished industrial micron films is inefficient, so it is necessary to reduce the pore size and increase the specific surface area, which in turn improves the catalytic effect.

### 3.2. CEC for the Production of H_2_O_2_

**Figure 6:**
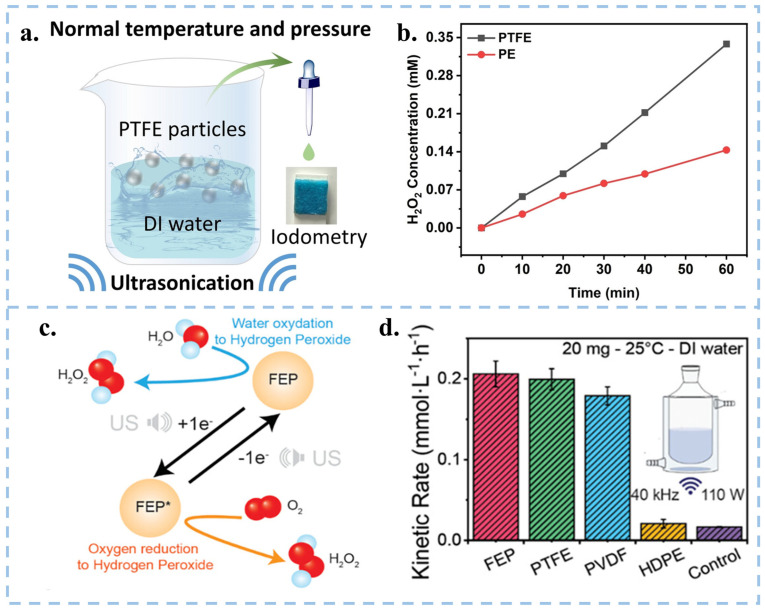
(**a**) Schematic diagram of the device for ultrasonic generation of H_2_O_2_ from PTFE particles in DI water. (**b**) Comparison of H_2_O_2_ yield prepared from PTFE and Polyethylene (PE) pellets under different time of sonication. Reproduced with permission from [20], Adv. Mater.; published by Wiley, 2023. (**c**) Schematic diagram of the mechanism of H_2_O_2_ generation by sonication of FEP powder in DI water. (**d**) Comparison of H_2_O_2_ yield of FEP, PTFE, PVDF and HDPE under ultrasonication. Reproduced with permission from [21], Nanoscale; published by RSC, 2023.

In addition to the degradation of organic substances, hydrogen peroxide (H_2_O_2_) was also generated by the CEC process involving solid catalysts of polymeric organics in DI water. In 2023, Wang et al. induced the generation of ·OH and ${\cdot O}_{2}^{-}$ via contact-separation of PTFE particles with the DI water/O_2_ interface under ultrasonication. This resulted in the subsequent generation of H_2_O_2_ through a series of reactions, achieving a yield as high as 313 µmol L^−1^ h^−1^ (Figure 6a,b) [20]. Subsequently, Wang and group used EPR and isotope labeling experiments to prove that the ultrasonic generation of H_2_O_2_ in DI water by FEP powder was based on the combination of ·OH or two ${\cdot O}_{2}^{-}$ (Figure 6c). Moreover, they compared the yield of H_2_O_2_ under ultrasonication among materials with high electronegativities (FEP, PTFE, and PVDF) and material without electron transfer ability (HDPE) [59], as shown in Figure 6d, HDPE hardly produces H_2_O_2_ after ultrasonication, so electron transfer between materials is the key to generating H_2_O_2_. To verify the formation of hydrogen peroxide, they used labeling experiments using H_2_^18^O to replace 20% of the volume of water. They further verified that H_2_O_2_ is actually produced by CEC triggered water oxidation (WOR) and oxygen reduction (ORR) reactions. Zare’s group O-atom isotope experiment also explore the origin of the oxygen atoms for the generation of H_2_O_2_. The observations suggest that the O atoms in H_2_O_2_ generation originate from surface hydroxyl groups on the SiO_2_ substrate [93]. The method of generating H_2_O_2_ using CEC not only does not require complex reaction systems and catalyst materials, but also does not produce harmful intermediates during the reaction process. This method has simple, mild, green, safe, and environmentally friendly reaction conditions, and has great potential for development. Given that powder-type catalysts are affected by factors such as particle size, concentration, reaction temperature, ultrasonic frequency, and power, there remains room for exploration in terms of the type and shape of catalysts for the synthesis of H_2_O_2_ via CEC.

### 3.3. CEC for the Recovery of Metals

The CEC mechanism is also used to recycle metals in electronic wastewater. In the development of electronic technology, the demand for precious metal materials has gradually increased. However, due to their rarity in the environment, researchers have developed methods to recover metals from electronic waste.

**Figure 7:**
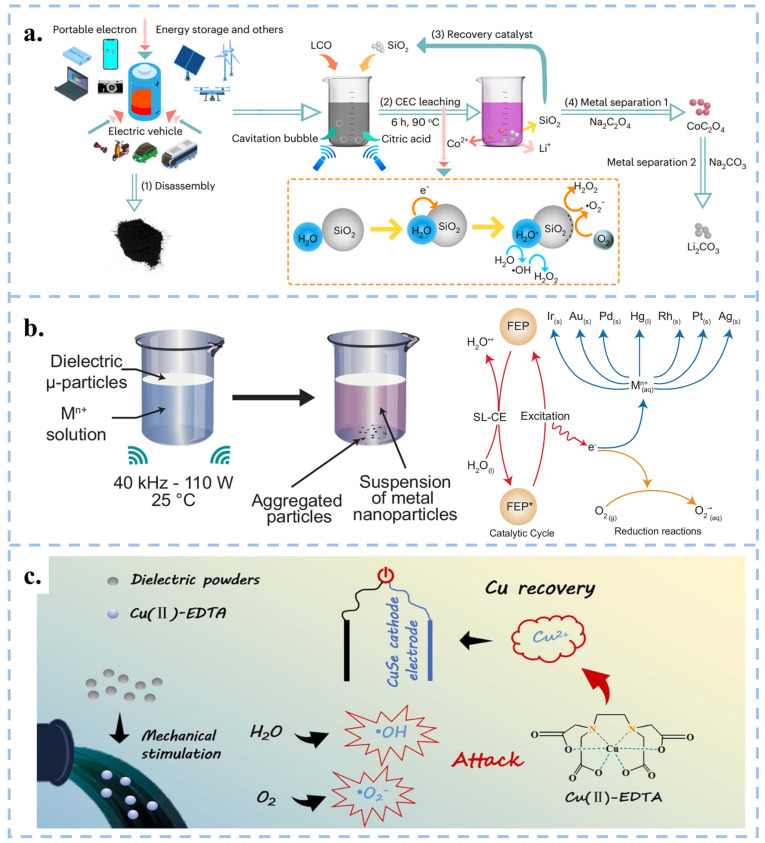
(**a**) CEC recycling flow chart of used lithium battery metal. Reproduced with permission from [94], Nat. Energy; published by Nature, 2023. (**b**) Experimental setup and process diagram for reducing various metal ions in aqueous solution using FEP driven by ultrasound. Reproduced with permission from [95], Nat. Commun.; published by Nature, 2024. (**c**) Process and schematic diagram of FEP powder ultrasonic degradation of Cu (II)-EDTA and recovery of Cu ions. Reproduced with permission from [96], J. Hazard. Mater.; published by Elsevier, 2024.

Compared to traditional methods of reducing metals, Wang et al. proposed a new approach using CEC instead of traditional reducing agents in organic acid leaching processes, which reduced lithium, cobalt, nickel, and manganese from discarded lithium batteries [94], and reduced precious metals at room temperature and pressure [95]. Specifically, recyclable Hydrophobic SiO_2_ powder served as a catalyst to stimulate continuous solid-liquid contact-separation through cavitation bubbles under ultrasonication, resulting in the continuous generation of ROS. The results revealed that in the case of lithium-cobalt (III) oxide batteries, the leaching efficiency reached 100% for Li and 92.19% for Co within 6 h at 90 °C. Meanwhile, for Li ternary batteries, the leaching efficiencies were 94.56% for Li, 96.62% for Ni, 96.54% for Mn, and 98.39% for Co at 70 °C (Figure 7a) [94]. FEP particles can be used to extract precious metal nanoparticles from aqueous solutions at room temperature, as well as gold from CPU leaching solutions and electroplating waste (Figure 7b) [95]. Similarly, Shen et al. achieved the degradation of Cu (II)-EDTA and the recovery of Cu^2+^ using FEP dielectric powder combined with capacitive deionization (CDI), with a degradation rate of 86.4% within 150 min (Figure 7c) [96]. Therefore, the CEC method provides a green, efficient, and economical approach for metal recycling, effectively meeting the exponential growth demand of industrial scale production.

### 3.4. CEC for Synthesizing Chemical Substances

**Figure 8:**
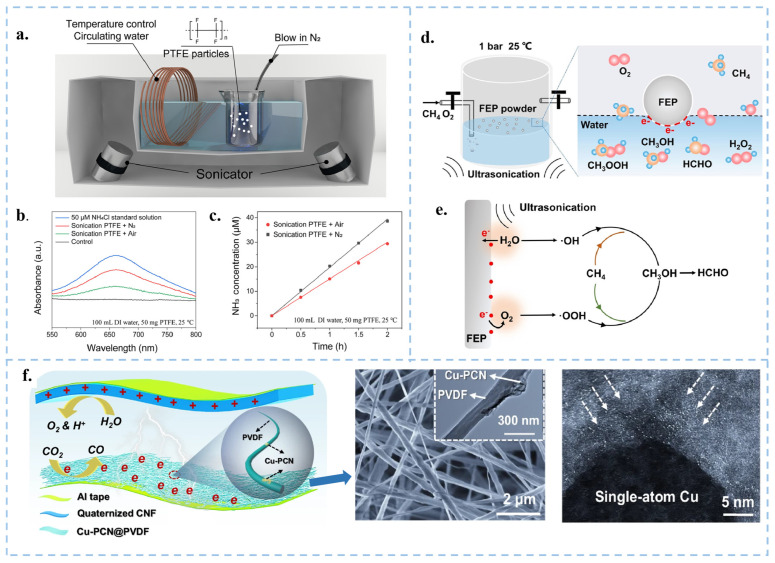
(**a**) Schematic representation of the experimental setup. (**b**) Absorbance variations using the indophenol blue method under sonication with different atmospheres. (**c**) Different ultrasonic times performed under the same conditions as in (**b**). Reproduced with permission from [104], Proc. Natl. Acad. Sci. USA; published by PNAS, 2024. (**d**) Schematic diagram of FEP powder ultrasonic oxidation methane experimental device. (**e**) CEC methane oxidation mechanism. Reproduced with permission from [105], Angew. Chem. Int. Ed.; published by Wiley, 2024. (**f**) Schematic diagram of CEC reducing CO_2_ in the air.106Reproduced with permission from [106], Nat. Commun.; published by Nature, 2024.

In 2019, Zare and colleagues discovered that many chemical reactions previously challenging to conduct in the liquid phase can spontaneously occur within microdroplets formed by carrier gas spray or ultrasonic atomization. Micro water droplets based on micrometer size exhibit strong interfacial electric fields and spontaneous charge transfer at the water–oil interface, leading to contact induced redox reactions [97]. Interestingly, the smaller the droplet size, the more pronounced these phenomena become [93,98]. Atomization of water induces charge separation, resulting in the generation of negatively and positively charged droplets. The surface charge of water droplets induces the formation of an electric double layer (EDL), rendering the droplets akin to electrochemical cells [99]. Water microdroplets exhibit various properties, including the presence of hydroxyl radicals (·OH) at the air–water interface [100], and oxidative coupling products of C-H/N-H [101]. Mehrgardi et al. achieved hydrogen peroxide production by bubbling ultrapure N_2_ through silica capillary tubes to form droplets, with the concentration of hydrogen peroxide found to increase when N_2_ was replaced by compressed air or O_2_ [102]. Song et al. demonstrated the generation of ammonia at the interface between water droplets and nitrogen gas by spraying water droplets onto iron oxide catalysts [103]. Li et al. utilized PTFE nanoparticles to facilitate continuous contact between water and solid particles in an ultrasound system under a nitrogen atmosphere, achieving continuous ammonia synthesis (Figure 8a–c) [104]. They observed that nitrogen introduction was more effective in synthesizing ammonia compared to air. The presence of trace oxygen in the air and dissolved oxygen in water increased the concentration of H_2_O_2_ while reducing the concentration of ammonia. Although several breakthroughs have been made in micro water droplets technology, the specific reaction mechanism still needs further in-depth exploration. In the future, theoretical tools such as quantum chemical calculations and molecular dynamics simulations can be used to reveal the dynamic process and mechanism of droplet chemical reactions at the microscopic level. In addition, it is particularly crucial to improve the droplet preparation technology, including but not limited to enhancing the preparation efficiency, enhancing the stability of droplets, and optimizing the uniformity of droplets, in order to adapt to more diverse chemical reaction requirements. At the same time, researchers should actively explore the integration of droplet technology with photocatalysis, electrocatalysis, and other catalytic methods, in order to develop more efficient and environmentally friendly new chemical reaction pathways. Ultimately, these efforts will help promote the wide application of droplet technology in the industrial sector and realize its great potential in the field of chemistry. Fan et al. used FEP powder to catalyze the oxidation of CH_4_ to HCHO and CH_3_OH through ultrasonic treatment (Figure 8d,e) [105]. Li et al. used CEC to reduce CO_2_ in the air and decrease its concentration [106]. They utilized electrospun polyvinylidene fluoride (PVDF) with a single copper atom-anchored polymeric carbon nitride (Cu-PCN) catalyst and quaternized cellulose nanofibers (CNF) as the triboelectric layers for triboelectric nanogenerators (Figure 8f). The quaternized CNF can effectively capture CO_2_ at low concentrations, while Cu-PCN can efficiently enrich electrons during the triboelectric charging process, facilitating the transfer of electrons upon contact with CO_2_ adsorbed on the quaternized CNF. This research advances CO_2_ reduction technologies, aligns with the development philosophy of CEC, and opens up a new direction for the application of CEC.

## 4. Design and Optimization of Catalytic Methods and Materials

At present, most of the catalyst materials used for CEC are high polymers such as FEP and PTFE. Table 2 lists the efficiency of degrading organic dyes by CEC and several other catalytic methods. It can be seen that compared with other catalytic methods, the catalytic efficiency of CEC needs to be improved. Therefore, we need to improve the catalytic efficiency by finding other ways or designing material structures, so that CEC can be truly used in industry. In the current research stage, the methods mainly used to improve the catalytic efficiency include CEC combined with other catalytic methods, adding functional groups, supplementing charges, etc.

### 4.1. Change of Catalytic Conditions

**Figure 9:**
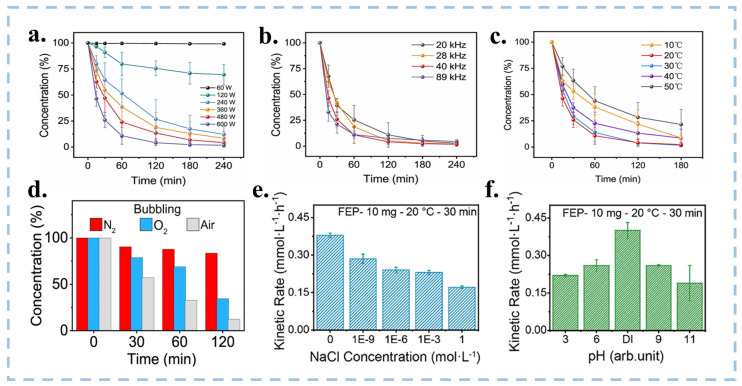
(**a**–**c**) Degradation of MO solution by FEP powder under different conditions (power, frequency and temperature). Reproduced with permission from [113], Nano Energy; published by Elsevier, 2022. (**d**) The influence of dissolved gas in solution on the degradation of MO. Influence of (**e**) the salt concentration (NaCl) and (**f**) the pH on the kinetic rate of the production of H_2_O_2_. Reproduced with permission from [21], Nanoscale; published by RSC, 2023.

The most common way to initiate CEC is ultrasonic. Wang et al. investigated the optimal parameters for CEC under varying reaction conditions, including different ultrasonic frequencies, powers, and temperatures [113]. The experimental results showed that the final degradation rate of the MO solution increases with the rise in ultrasonic power. The highest final degradation rate is observed at an ultrasonic frequency of 40 kHz and a power of 600 W, as depicted in Figure 9a,b. This is because higher power and frequency can excite more cavitation bubbles, causing more frequent contact separation and promoting electron transfer at the solid–liquid interface. Notably, the optimal temperature for CEC occurrence is found to be between 20–30 °C (Figure 9c); this is due to the FEP powders having a glass transition at high temperatures [113]. Nevertheless, large-scale ultrasonication consumes significant energy and generates a lot of heat when treating wastewater in industrial settings, making it difficult to maintain room temperature. This is not conducive to catalytic reactions and environmental protection. In the future, we need to search for catalysts that can perform CEC reactions under extreme conditions. Different atmospheric conditions can affect the catalytic effect. Wang’s team [20,21,113] conducted experiments on the degradation of MO and the production of H_2_O_2_ under the atmospheric conditions of air, O_2_, and N_2_, respectively, as shown in Figure 9d. Both the introduction of O_2_ and N_2_ would reduce the reaction rate of CEC, indicating that electron exchange occurs between the interface of oxygen and the catalyst due to CE. However, an oxidizing atmosphere is detrimental to the contact electrification between materials, and a high concentration of O_2_ can affect the transfer of electrons. The salt concentration and pH value in the solution will also affect the CEC reaction. As shown in Figure 9e,f, the higher the concentration of acid, base, and salt, the lower the reaction rate, so CEC is more suitable for reaction under neutral conditions [21]. Apart from environmental factors, the properties of the catalyst itself also have a significant impact on CEC, such as the amount of catalyst added and its particle size. The catalysts used in CEC are generally hydrophobic, and powdered catalysts are prone to agglomeration in water, making it difficult for them to disperse. This results in a reduced surface area in contact with the aqueous solution, which in turn decreases the reaction rate. Therefore, we need to maintain appropriate conditions during the CEC process and continuously explore CEC that can be performed under extreme conditions.

### 4.2. Coupling of CEC with Other Catalytic Methods

**Figure 10:**
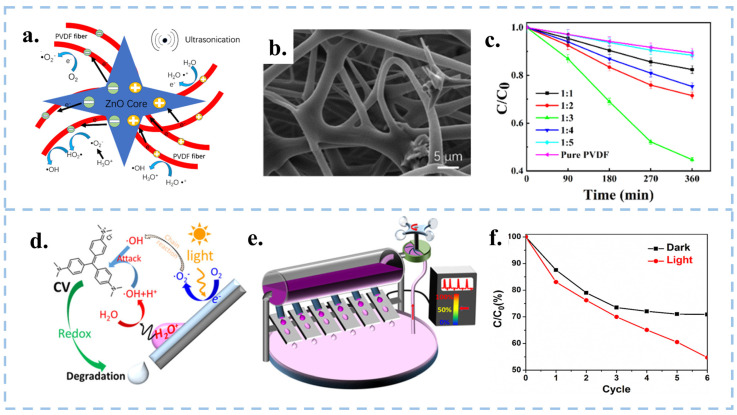
(**a**) Schematic diagram of the contact piezoelectric biocatalytic mechanism of ZnO@PVDF composite membrane. (**b**) SEM image of ZnO:PVDF composite film. (**c**) Concentration of ZnO:PVDF composite membranes degrading MO as a function of time. Reproduced with permission from [114], Molecules; published by MDPI, 2022. (**d**) Schematic diagram of the CEC mechanism of a droplet sliding down a slope. (**e**) Wind-driven multi-droplet CEC systems. (**f**) Changes in absorbance of CV under dark and light conditions. Reproduced with permission from [30], Water Res.; published by Elsevier, 2022.

CEC utilizes mechanical energy from the environment for catalysis, making it well-suited for combination with other catalytic methods such as photocatalysis and piezocatalysis, in order to enhance reaction rates. Zhang et al. applied the electrospinning technique to fabricate ZnO@PVDF composite films (Figure 10a–c), which combined CEC with the piezoelectric catalysis of tetrapod ZnO and the β-phase polyvinylidene difluoride (PVDF), resulting in a remarkable 444.23% increase in catalytic degradation efficiency compared to CEC (pure PVDF film) [114]. The design of the ZnO@PVDF composite film combines piezoelectricity and the ability of CEC, and the synergistic effect interaction between these two materials prolonged the existence time of electron-hole pairs and provided favorable conditions to generate ·OH, and then, improves the catalytic efficiency of the PVDF film. Organic-inorganic composite films can combine the characteristics of inorganic and organic materials through techniques such as electrospinning, and increase surface potential by doping different forms of inorganic nanomaterials into organic materials. However, the selection of composite membrane materials and the design of structures require in-depth exploration.

The combination of photocatalysis and CEC was proposed by Long et al. Under the condition of light illumination, the droplets slipping on the inclined surface produced an ultra-high degradation rate [30]. As depicted in Figure 10d–f, electrons on the PTFE surface gradually accumulated in the dark, eventually reaching saturation. At this point, the water molecules in the solution only produced ·OH, causing the CEC process to cease after a certain period of time. Contrastingly, in the presence of light, the energy of photons absorbed by electrons on the film surface is more likely to overflow, which subsequently combined with O_2_ in the water to form ${\cdot O}_{2}^{-}$, possessing high oxidizing properties. The illumination can compensate for the electron saturation in the CEC process in this mode and greatly improve the catalytic efficiency. Apart from that, it is also possible to combine tribocatalysis with CEC. For example, with simultaneous stirring and sonication, electrons can be both leaped in solid-solid friction and exchanged between solid-liquid. The simultaneous presence of electron leaps and electron transfer improves the degradation efficiency of the system. CEC can work together with other catalytic methods to improve the catalytic efficiency, and there are many more combinations to be explored, for example, the combination of thermal catalysis or magnetic field and CEC.

### 4.3. Surface Modification and Functionalization of Catalysts

**Figure 11:**
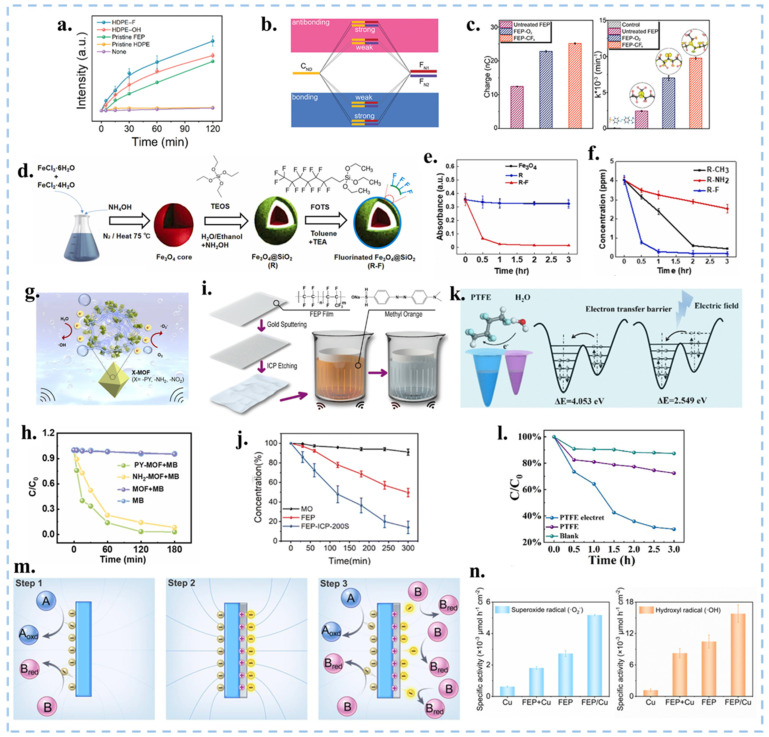
(**a**) UV-Vis absorbance intensity of WST-1 formazan dye during different ultrasonic with functional group modified membrane. Reproduced with permission from [115], Nano Res.; published by Springer, 2024. (**b**) Schematic diagram depicting the influence of the strength of orbital hybridization strength on the energy of the hybrid orbitals. (**c**) The charge output and kinetic rate of MO degradation of untreated FEP, FEP-O_2_, and FEP-CF_4_. Reproduced with permission from [28], Adv. Funct. Mater.; published by Wiley, 2024. (**d**) Synthesis process of magnetic Fe_3_O_4_@SiO_2_-F nanoparticles. (**e**,**f**) CEC performance under different conditions (material, functional group) for the degradation of MO. Reproduced with permission from [116], Nano Energy; published by Elsevier, 2023. (**g**) Degradation process of PY-MOF in water. (**h**) Concentrations of MOF and MOF (-PY, -NH_2_) degrading MB with time. Reproduced with permission from [121], Nano Energy; published by Elsevier, 2023. (**i**) Device and process of FEP film CEC degradation of MO and SEM image of FEP films etched by ICP for different times. (**j**) Concentration of ultrasonically degraded MO in membranes after FEP and ICP etching over time. Reproduced with permission from [22], Nat. Commun.; published by Nature, 2024. (**k**) Schematic diagram of the principle of a polarizer. (**l**) Comparison of the roles of polytetrafluoroethylene (PTFE) and the internal electric field in MO dye degradation. Reproduced with permission from [122], J. Am. Chem. Soc.; published by ACS, 2024. (**m**) Schematic diagram of the catalytic principle of the polymer/metal Janus composite catalyst. (**n**) The amount of free radicals generated by metal, polymer, metal-polymer, and polymer/metal Janus composite catalysts, respectively. Reproduced with permission from [123], Nano Energy; published by Elsevier, 2023.

The strong electron-carrying ability of the fluorine functional groups was the main factor contributing to the efficacy of the CEC process. In 2023, Tang et al. delved into exploring the role of surface functional groups in the CEC process [115]. They investigated the impact of -F and -OH functional groups in CE during solid-liquid interaction using high-density polyethylene (HDPE) membranes characterized by poor charge transfer capacity. The charge recovery of HDPE-F was 2.5–2.7 times greater than that of the HDPE-OH, with catalytic activity proving to be 2.4 times higher compared to commercial fluorinated membranes (Figure 11a). In order to investigate the sources and locations of electron transfer in depth and develop methods for controlling electron transfer, Wang et al. discussed the effect of electronic defects on the CEC capability of high-performance friction materials, such as fluorinated polymers. Both theory and experiment have verified that defect passivation alters the band edge of FEP, eliminates deep defect states, and makes electron transfer easier. The Crystal Orbit Hamilton Population Project (COHP) located the positions of deep defect states before and after defect passivation (Figure 11b), and it can be seen that the hybridization intensity weakened from low-energy bond states to high-energy bond states after passivation. The experimental results of Figure 11c have verified that with the introduction of functional groups, such as -F, =O, -CF_3_, to replace different types of defects, it was observed that the kinetic rate of degradation of MO by CEC increased by 398% when using a passivated FEP film. This indicates that the positions of the highest occupied molecular orbital (HOMO) and deep level defect states are key to the CE and CEC capabilities [28]. This study can provide a theoretical basis for designing high-performance CEC materials. Surface modification with specific functional groups can enhance CEC capability, but its internal reasons need further investigation. Liu et al. proposed the use of SiO_2_-coated magnetic Fe_3_O_4_ nanoparticles as a catalyst substrate. This approach improves the efficiency of CEC degradation of MO by modifying the chemical groups on the surface to increase electronegativity (Figure 11d). Additionally, the incorporation of magnetic particles simplifies the recycling process of a catalyst via magnetic force, thereby effectively improving the recovery rate [116]. The degradation efficiency of MO exhibited a notable increase corresponding to the ascending electronegativity of chemical groups (R-F > R-CH_3_ > R-NH_2_), as illustrated in Figure 11e,f. Metal-organic frameworks (MOFs) assembled from organic ligands and metal junctions [117] exhibit high specific surface areas [118], designable topology [119], and tunable semiconductor features [120]. In the realm of TENGs, MOFs have been employed to enhance the output or expand various new applications. In this direction, Guo et al. modified MIL-101(Cr) with a pyridine (-PY) group, showing positive frictional electrical properties during contact-separation with PTFE (Figure 11g). The cationic dye methylene blue (MB) solution underwent degradation through CEC with PY-MOF, accelerating the chemical reaction to form ·OH [121].

As illustrated in Figure 11h, the addition of 20 mg PY-MOF powders to a 50 mL 20 mg L^−1^ MB solution resulted in a remarkable 97% degradation within 3 h. This work validated that the modification of MOF molecules with functional groups can increase the positive frictional electrical properties of MOFs. This stands in contrast to previous studies focusing on electronegative materials in CEC, thereby expanding the spectrum of catalysts with great potential for various applications. In addition to grafting functional groups to catalysts, Wang et al. modified the FEP film by inductively coupled plasma (ICP) etching with Argon (Ar) to form a conical micro-nanostructure on the surface of the film (Figure 11i,j), thereby increasing the hydrophobicity and specific surface area of the film. Due to the lower electron affinity at the top of the nano cone, the electron transfer ability can be effectively enhanced, thus improving its degradation efficiency in CEC, confirming the importance of the microstructure design on the membrane surface [22]. This work explores the effect of the microstructure of the film surface on the CEC efficiency and establishes a foundation for film-based processes. The use of FEP and PTFE membrane materials improved the recovery rate and recyclability, but the degradation efficiency of the membrane without ICP modification was lower than that after modification, which is related to the microstructure of the membrane surface. To enhance the electronic ability of the surface and improve the catalytic efficiency, we can modify the surface structure of the membrane, such as introducing micro/nanostructures, impurities or defect passivation. The addition of surface functional groups aims to enhance the catalyst’s ability to extract electrons from water. However, there are two main processes involved in the redox reaction catalyzed by CEC: the water oxidation reaction (WOR) and the oxygen reduction reaction (ORR). We can regulate the rate of the catalytic reaction by adjusting the intensity of these two processes individually. Based on the principle that carrier separation can enhance the photocatalytic and piezoelectric catalytic performance of ferroelectric materials, Zhang et al. charged inert polymeric dielectrics (e.g., PTFE) through the electret process, thereby establishing an internal electric field that significantly improved the degradation efficiency of MO (Figure 11k,l) [29]. This demonstrates that an electric field can facilitate electron transfer between the solid-liquid interface. Consequently, we can enhance the catalytic efficiency of CEC by charging it. Tang et al. deposited metal onto a polymer through magnetron sputtering to form a polymer/metal Janus composite catalyst. The reaction rates of WOR and ORR can be adjusted by modulating the derivative electric field generated by the polymer, as well as the work function and conductivity of the metal [122]. The accumulation of CE charges on the surface of the polymer generates a derivative electric field, which can lower the energy barrier for electron transfer on the metal surface, thereby facilitating the release of electrons from the metal (Figure 11m,n). It’s interesting that by selecting polymers with opposite polarity, the rates of specific reaction pathways in existing metal-based catalytic systems can be regulated. Therefore, it can be concluded that establishing an internal electric field is conducive to enhancing catalytic efficiency. These studies have, for the first time, regulated the reaction pathways of CEC, pushing it to a new stage.

### 4.4. Changing the Way of CEC Startup

**Figure 12:**
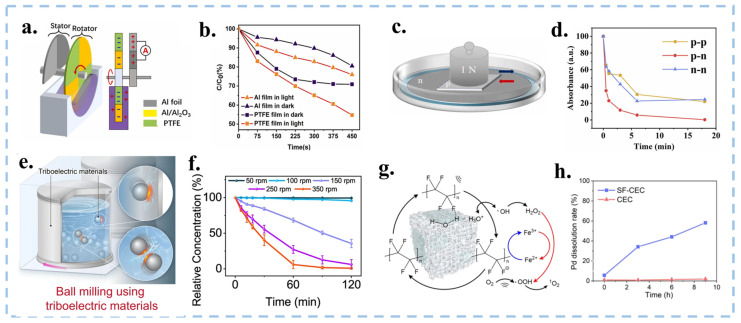
(**a**) Rotation mode degradation device. (**b**) Degradation efficiencies of CV of PTFE and Al films under dark and light conditions. Reproduced with permission from [123], Nano Energy; published by Elsevier, 2023. (**c**) Schematic diagram of the device structure for MB degradation using the tribovoltaic effect. (**d**) The absorbance changes of MB at different times when different types and the same type of silicon wafers friction. Reproduced with permission from [124], Nano Energy; published by Elsevier, 2024. (**e**) Schematic diagram and working principle of ball milling process using triboelectric materials. (**f**) Evolution of relative concentration of MO in different revolution speeds. Reproduced with permission from [23], J. Clean. Prod.; published by Elsevier, 2020. (**g**) Schematic diagram of the SF-CEC system. (**h**) The dissolution performance of precious metals in Pd-ZnTiO_3_ using SF-CEC and CEC. Reproduced with permission from [125], Angew. Chem. Int. Ed.; published by Wiley, 2024.

In life, there are many forms of mechanical energy. As a type of mechanical catalysis, CEC ideally should make the best use of green and simple mechanical energy from the environment. Since the TENGs have four modes of friction, Long et al. have designed a slope mode, a rotation mode, and a solid-liquid-solid mode based on the Lateral-Sliding Mode and Single-Electrode Mode. Among them, the slope mode was achieved by rolling down crystal violet (CV) droplets on a PTFE dielectric membrane [30].The rotation mode design is an Al@Al_2_O_3_/PTFE membrane covered disk rotating in solution (Figure 12a,b) [123]. The solid-liquid-solid mode combined with tribovoltaic effect degrades methylene blue solution by friction of n-type and p-type silicon wafers (Figure 12c,d) [124]. These approaches not only significantly improve the catalytic efficiency of a single material but also make full use of clean energy sources such as wind and water. Also, these modes can output electrical signals to facilitate real-time monitoring of degradation levels.

Ball milling is a representative mechanochemical strategy. As shown in Figure 12e,f, Wang et al. designed a liquid assisted grinding method using PTFE material balls and bottles to achieve CEC degradation of MO solution [23]. The higher the rotational speed, the more frequent collisions occur, achieving stronger electron cloud overlap and reducing the energy barrier for electron exchange. However, there is a speed threshold that triggers CEC. The ball milling method of CEC is expected to use more frictional materials for mechanochemical catalysis under mild conditions in the future. Under neutral conditions, some of the ·OH will combine to form H_2_O_2_ without decomposing, which limits the utilization of ·OH and catalytic efficiency. A novel Fe^III^-initiated self-cycling Fenton reaction into CEC (SF-CEC) was proposed by Fan et al., which introduced Fe^III^ to promote the decomposition of H_2_O_2_ under acidic conditions, thereby achieving a rate six times greater than that of traditional CEC for degrading azo dyes (Figure 12g,h) [125]. The system initiates a self-circulating Fenton reaction upon the introduction of Fe^III^, activating H_2_O_2_, which then reduces Fe^III^ to Fe^II^. Subsequently, Fe^II^ is oxidized back to Fe^III^ by electrons on the surface of PTFE, forming a self-circulating CEC. This process enables rapid degradation of MO under acidic conditions. This work provides CEC with a novel approach, enabling it to react under acidic conditions and offering new insights for the future development of CEC.

## 5. Summaries and Perspectives

The application of the principle of SL-TENGs to catalysis by CEC represents a novel approach to produce reactive oxygen species. This catalytic method can collect mechanical energy under natural conditions to facilitate chemical reactions. It has been successfully utilized in the degradation of organic molecules, synthesis of chemical substances, and reduction of metals. Unlike conventional catalytic methods, CEC utilizes various forms of mechanical energy, including wind and water, and other renewable energy sources, enabling it to react under natural conditions without relying on specific energy inputs such as light or electricity. Compared with other catalytic methods, the occurrence of CEC is extremely convenient. As long as the material can come into contact with electricity, catalytic reactions can be achieved. Therefore, this greatly expands the selection range of catalysts and provides the possibility for designing more diverse catalytic systems. Moreover, compared to methods such as lighting and electrical energy input that determine local reaction effects, the reaction range of CEC is more comprehensive and has the potential for large-scale applications. In addition, CEC has the unique advantage of being green and environmentally friendly, thanks to its almost unlimited selection of catalysts. Therefore, a large number of environmentally friendly materials can be used for catalysis, and these catalysts can be efficiently separated from substrates in a simple way. This not only avoids secondary pollution to the environment but also allows for repeated recycling and use. However, the limited variety of catalysts currently developed by CEC, and most CECs rely on ultrasound and stirring processing, has the problem of long reaction time and low catalytic efficiency. Moreover, the chemical reaction behavior and mechanism of CEC are still unclear. From our perspective, future research on CEC should prioritize the following areas:

(1) **Urgent need to explore novel materials for CEC:** The predominant catalysts used in CEC are fluorinated polymer powders, with a minimal portion being inorganic materials modified with fluorine or other functional groups. This limited variety in catalyst types raises concerns about potential environmental pollution from fluorides. There is a critical need to explore and develop new materials to expand the catalyst repertoire. Advanced computational techniques such as artificial intelligence and machine learning can play a crucial role in effectively screening;
(2) **Exploring the mechanism of CEC:** There is an urgent need to delve deeply into the intricate electrocatalytic mechanisms within CEC systems. Currently, there is a notable absence of standardized methodologies for accurately assessing the electronic states associated with frictional charges in insulating materials like polymers. Given the unique degradation barriers of each organic dye, it is imperative to undertake comprehensive theoretical investigations into the charge transfer kinetics, and catalytic reaction kinetics under varying catalytic environments of contact electrocatalysis. Additionally, the development of sophisticated in-situ characterization techniques is paramount, including advanced surface spectroscopic methods and in-situ characterization techniques to study CEC mechanism, which have yet to be comprehensively addressed in the existing literature;
(3) **Exploring the structure of CEC materials:** Optimizing surface morphology and band structure is expected to improve catalytic performance. Introducing doping and defects can alter the work function of catalysts, thereby increasing the amount of electron transfer. Designing construct hydrophobic surfaces can reduce lateral friction and charge dissipation. Introducing micro/nanostructures to increase contact surface area can improve catalytic efficiency. Theoretically, all materials capable of electron exchange with liquids hold promise for use in CEC, offering an exciting prospect for exploring the structure of the materials;
(4) **Designing novel operational modes for CEC:** Implement innovative operating models, combine different catalytic methods, integrate wind energy, solar energy, potential energy, thermal energy, and magnetic energy storage, and improve catalytic efficiency;
(5) **Developing standards and novel applications of CEC:** CEC is currently at a preliminary stage of development within laboratory environments. The lack of standardized experimental protocols and comprehensive data reporting protocols poses challenges to the reproducibility and comparability of experimental results. Moreover, potential issues in scalability, energy conversion efficiency or material degradation over time need to be addressed. Therefore, it is imperative to establish rigorous standards and guidelines in laboratory practices, facilitating accurate and equitable assessments of catalytic performance across various research facilities. Furthermore, the progression of CEC hinges on collaborative efforts spanning multiple disciplines, including materials science, electrochemistry, fluid dynamics, physics, and environmental engineering. At present, CEC has demonstrated multifaceted capabilities including organic molecules degradation, H_2_O_2_ production, metal recovery, depending on specific solution compositions and gas environments. This versatility provides the possibility for its future expansion into diverse fields such as eco-friendly chemical synthesis, biomedical applications, and environmental remediation. Potential applications include but are not limited to water splitting, tooth whitening, pharmaceutical loading, heavy metal reduction, bio-inspired and enzyme-mimetic catalysts, etc.

## Figures and Tables

**Table 1:** Characteristics of common catalysts used for electrocatalysis, photocatalysis, piezoelectric catalysis, tribocatalysis and CEC, as well as their advantages and disadvantages.

Catalytic Mode	Characteristics	Advantages	Disadvantages
Electrocatalysis	Precious metal (Pt, Ru, Ir), and metal oxides, etc. [15,16].	Good stability and catalytic activity	Need external electric field, high energy consumption
Photocatalysis	Metal oxide, metal sulfide and other semiconductor materials, etc. [13,14].	High oxidation efficiency and low cost	Short life of catalyst, limited light-responsive wavelength range and needs light excitation
Piezoelectric Catalysis	A metal or semiconductor material having piezoelectric properties [77,78]	Environmental protection, without external excitation and light sources	Limited selection of materials
Tribocatalysis	Combination of semiconductor materials and high electronegativity materials [60]	Wide selection of materials	Low catalytic efficiency
Contact-electro-catalysis	Dielectric material with high electronegativity [28]	Wide selection of materials and applications	Need to explore types of catalysts

**Table 2:** CEC and other catalytic methods used to degrade organic substances and their catalytic efficiencies.

Catalysts	Catalyst Characteristics	Dye Species	Dye Concentration (mg/L)	Form Mechanical Action	Catalytic Activity	Ref.
FEP Powders	Contact-electro-catalysis	MO	5	Ultrasonication (120 W/40 kHz)	~98.1%, 180 min	[17]
(CF_x_)_n_	Contact-electro-catalysis	MO	5	Ultrasonication (600 W/45 kHz)	~95%, 180 min	[89]
PTFE	Contact-electro-catalysis	TET	5	Ultrasonication	~90%, 90 min	[90]
ZnO/CuO	Photocatalysis	CV	5	UV–vis light irradiation	~90%, 120 min	[107]
SnO_2_	Photocatalysis	MB	10	175W high-pressure mercury lamp irradiation (365 nm)	~90%, 50 min	[108]
MoSe_2_ nanoflowers	Piezoelectric catalysis	RhB	10	Ultrasonication (250 W/40 kHz)	~90%, 0.5 min	[109]
SrFeO_3−x_	Piezoelectric catalysis	TC	30	Ultrasonication (150 W/40 kHz)	~96%, 75 min	[8]
CdS nanowires	Tribocatalysis	RhB	5	Stirring (300 rpm)	~98%, 420 min	[110]
BaSrTiO_3_ nanoparticle	Tribocatalysis	RhB	5	Stirring (300 rpm)	~99%, 180 min	[111]
TiO_2_ nanoparticles	Tribocatalysis	MB	20	Stirring (400 rpm)	~94.7%, 180 min	[112]

MO: Methyl orange, MB: Methylene blue, CV: Crystal violet, RhB: Rhodamine B, TC: Tetracycline. TET: Tetracycline.

## Data Availability

Data is contained within the article.
